# Novel PHEX gene locus‐specific database: Comprehensive characterization of vast number of variants associated with X‐linked hypophosphatemia (XLH)

**DOI:** 10.1002/humu.24296

**Published:** 2021-12-05

**Authors:** Soodabeh Sarafrazi, Sean C. Daugherty, Nicole Miller, Patrick Boada, Thomas O. Carpenter, Lauren Chunn, Kariena Dill, Michael J. Econs, Scott Eisenbeis, Erik A. Imel, Britt Johnson, Mark J. Kiel, Stan Krolczyk, Prameela Ramesan, Rebecca Truty, Yves Sabbagh

**Affiliations:** ^1^ Medical Affairs Ultragenyx Pharmaceutical, Inc. Novato California USA; ^2^ Department of Pediatrics (Endocrinology) Yale University School of Medicine New Haven Connecticut USA; ^3^ Data Science Genomenon Inc. Ann Arbor Michigan USA; ^4^ Division of Endocrinology and Metabolism Indiana University School of Medicine Indianapolis Indiana USA; ^5^ Medical Affairs Invitae Corporation San Francisco California USA; ^6^ External Relations Invitae Corporation San Francisco California USA; ^7^ Research and Development Inozyme Pharma Boston Massachusetts USA

**Keywords:** fibroblast growth factor 23 (FGF23), locus‐specific database, osteomalacia, Phosphate regulating gene with Homology to Endopeptidases that maps to the X chromosome (PHEX), rickets, X‐linked hypophosphatemia (XLH)

## Abstract

X‐linked hypophosphatemia (XLH), the most common form of hereditary hypophosphatemia, is caused by disrupting variants in the *PHEX* gene, located on the X chromosome. XLH is inherited in an X‐linked pattern with complete penetrance observed for both males and females. Patients experience lifelong symptoms resulting from chronic hypophosphatemia, including impaired bone mineralization, skeletal deformities, growth retardation, and diminished quality of life. This chronic condition requires life‐long management with disease‐specific therapies, which can improve patient outcomes especially when initiated early in life. To centralize and disseminate *PHEX* variant information, we have established a new *PHEX* gene locus‐specific database, *PHEX* LSDB. As of April 30, 2021, 870 unique *PHEX* variants, compiled from an older database of *PHEX* variants, a comprehensive literature search, a sponsored genetic testing program, and XLH clinical trials, are represented in the *PHEX* LSDB. This resource is publicly available on an interactive, searchable website (https://www.rarediseasegenes.com/), which includes a table of variants and associated data, graphical/tabular outputs of genotype‐phenotype analyses, and an online submission form for reporting new *PHEX* variants. The database will be updated regularly with new variants submitted on the website, identified in the published literature, or shared from genetic testing programs.

## Graphical abstract

X‐linked hypophosphatemia (XLH), the most common form of hereditary hypophosphatemia, is caused by disrupting variants in the PHEX gene. To centralize and disseminate PHEX variant information, we have established a new PHEX gene locus‐specific database, PHEX LSDB, which presently describes 870 unique PHEX variants. This resource is publicly available on an interactive, searchable website (https://www.rarediseasegenes.com/) and will be updated regularly with new variants submitted on the website, identified in the published literature, or shared from genetic testing programs. (Ribbon diagram of the human PHEX protein generated in PyMol based on structures of homologous endopeptidases [Kelley et al., 2015]).

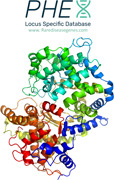

## 1 INTRODUCTION

Hypophosphatemic rickets (HR) was originally described in 1937 as a form of childhood rickets unresponsive to vitamin D in doses that were typically effective for the treatment of nutritional rickets (Albright et al., 1937). In contrast to transient nutritional deficiency, patients identified with this disorder were found to occur in families and experienced lifelong chronic hypophosphatemia due to excessive renal phosphate loss. Subsequent investigations have discovered that the proximal cause of the impaired renal retention of phosphate is increased serum levels of fibroblast growth factor 23 (FGF23), a phosphate regulating hormone expressed in bone by osteocytes and osteoblasts (Beck‐Nielsen et al., 2019). X‐linked hypophosphatemia (XLH; MIM# 307800), the most common form of hereditary HR, is caused by variants in the *PHEX* gene located at Xp22.1 (Francis et al., 1995). XLH shows X‐linked inheritance and affects ~1/20,000 males and females of all ages globally (Beck‐Nielsen et al., 2010).

### 1.1 PHEX and the XLH disease pathway

**Figure 1:**
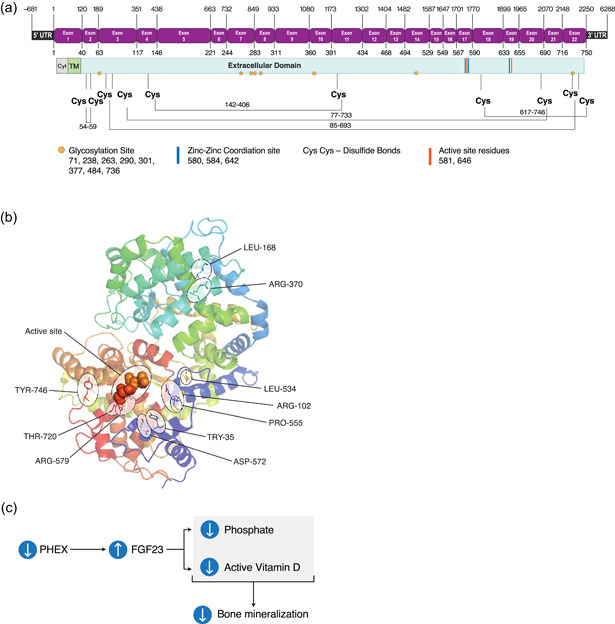
PHEX and the XLH disease pathway. (a) PHEX exon (top) and protein (bottom) maps indicating the positions of UTR boundaries, intron/exon junctions, and key functional/structural elements. Nucleic acid positions are indicated above the exon map and amino acid positions below. Cys, cystine; Cyt, cytoplasmic region; TM, transmembrane domain. (b) Ribbon diagram of the human PHEX protein generated in PyMol based on structures of homologous endopeptidases (Kelley et al., 2015); labels call out the enzymatic active site and amino acids altered in the 10 most common missense variations. (c) In the XLH disease state, decreased PHEX activity leads to an increase in serum FGF23, which decreases blood phosphate levels due to increased renal phosphate wasting. FGF23 also decreases the synthesis and increases the metabolism of the active vitamin D metabolite, ultimately leading to reduced bone mineralization. PHEX, Phosphate regulating gene with Homology to Endopeptidases that maps to the X chromosome; XLH, X‐linked hypophosphatemia; UTR, untranslated region

*PHEX* (Phosphate regulating gene with Homology to Endopeptidases that maps to the X chromosome) belongs to a well‐defined family of zinc metalloendopeptidases involved in cancer, bone‐renal diseases, cardiovascular disease, Alzheimer's disease, arthritis, and inflammatory disorders (Rowe, 2004; Turner & Tanzawa, 1997). The human *PHEX* gene encodes a 749 amino acid type II single integral transmembrane protein with most of the protein forming a large extracellular domain that contains the enzymatic active site, three zinc coordination sites, and multiple glycosylation sites and disulfide bonds (Figure 1a,b).

Multiple mouse models that harbor pathogenic variants in *PHEX* have been used to study XLH and its potential treatments. These include Hyp mice, which contain a 58 kb deletion of the 3′‐end of the gene extending into the untranslated region (UTR) (Sabbagh et al., 2002), Gy mice, which contain a large deletion that encompasses *PHEX* exons 1–3 and also extends into the upstream gene (Lorenz et al., 1998), Ska1 mice, which contain a single nucleotide variant in a splice donor site near the 5′‐end of the gene (Carpinelli et al., 2002), Jrt mice, which contain a stop codon at amino acid 496 (Owen et al., 2012), and Hyp‐2J and Hyp‐Duk mice (Han et al., 2012), which contain frameshift deletions affecting exons 13–14 and exon 15, respectively. The phenotypes of these mice vary somewhat depending on the background strain, but they all recapitulate the clinical features of XLH (hypophosphatemia, elevated serum FGF23, rickets and osteomalacia) and have been instrumental in elucidating the molecular basis of the disease.

The pathogenesis of phosphate wasting and impaired mineralization in individuals with XLH is generally attributed to inappropriately increased FGF23 levels (Figure 1c). *PHEX* is predominantly expressed in osteoblasts and osteocytes and is hypothesized to play a role in the phosphate sensing mechanism (Beck‐Nielsen et al., 2019). Although the molecular mechanism by which PHEX influences FGF23 levels is not known, it is clear that inactivating *PHEX* variants lead to increased *FGF23* expression and increased circulating FGF23, which causes renal phosphate wasting, decreased synthesis of the active metabolite of vitamin D (1,25‐dihydroxyvitamin D), and enhanced metabolism of 1,25‐dihydroxyvitamin D to 1,24,25‐trihydroxyvitamin D (Beck‐Nielsen et al., 2019). Although the physiological substrate for PHEX is not yet know, variants that disrupt the folding, trafficking, or enzymatic function of the protein have all been linked to the development of XLH in patients.

PHEX may also have important functions independent of phosphate regulation through FGF23. In vitro assays have demonstrated that PHEX can directly cleave osteopontin (OPN), an extracellular matrix protein found in bone and teeth and a potent inhibitor of mineralization (Barros et al., 2013; Qin et al., 2004). In mouse models with *PHEX* inactivating variants, full‐length OPN accumulates in the matrix of bone and is thought to contribute to impaired mineralization (Barros et al., 2013).

### 1.2 XLH clinical presentation and diagnosis

XLH patients typically begin to show signs of the disorder by 1–2 years of age and present clinically with growth retardation, rickets/bone deformity, and gait abnormalities (Haffner et al., 2019; Pavone et al., 2014). Pediatric patients have also demonstrated osteomalacia, craniosynostosis, and dental abscesses in otherwise healthy teeth (Haffner et al., 2019; Pavone et al., 2014; Reid et al., 1989). In addition to consequences of the progressive accumulation of skeletal defects (e.g., short stature, lower limb deformities, and osteoarthritis), adult patients experience ongoing disease manifested by impaired muscle function, pseudofractures, dental complications, enthesopathy (mineralization of ligaments and tendons), osteoarthritis and hearing loss (Beck‐Nielsen et al., 2009). In both children and adults, XLH symptoms can include chronic pain and functional disability, leading to a diminished quality of life (Dahir et al., 2020).

A definitive diagnosis of XLH is established with the combination of clinical and family history, physical examination, radiographic and laboratory findings, and often, genetic testing. Clinical and radiographic findings of rickets combined with laboratory findings of hypophosphatemia, and increased renal phosphate clearance are highly indicative of HR. XLH, and most other etiologies of familial HR, also exhibit low‐normal levels of circulating 1,25‐dihydroxyvitamin D. The ratio of renal tubular maximum reabsorption rate of phosphate to glomerular filtration rate (TmP/GFR) is reduced and circulating intact FGF23 levels are usually elevated or at least higher than the normal population mean. Information regarding potentially affected family members can help to determine the underlying cause of HR, but sporadic occurrences are common and other genetic causes are possible. A definitive genetic diagnosis of XLH requires confirmation of a pathogenic variant in the *PHEX* gene (Carpenter et al., 2011; Haffner et al., 2019).

### 1.3 PHEX variants genotype and phenotype

Pathogenic variants in human *PHEX* have been identified throughout the entire length of the gene and include frameshift, splicing, copy‐number, nonsense, and missense variants (Beck‐Nielsen et al., 2019; Sabbagh et al., 2000). These variants are predicted to cause loss of function protein changes, with the majority (>70%) producing a truncated PHEX protein. Studies of recombinant PHEX proteins in human cell culture assays have shown that missense variants can impair protein function by disrupting cellular processing, endopeptidase activity, or protein conformation (Li et al., 2020; Sabbagh et al., 2003, 2001; Zheng et al., 2020).

Controversy exists regarding potential correlation between *PHEX* genotypes and the severity of XLH phenotypes. It has been reported that female patients with certain variations in the *PHEX* gene may have less severe hypophosphatemia and milder skeletal deformity compared to males with the same *PHEX* variants, and some reports have suggested that patients with truncating variants or variants in the C‐terminal half of the *PHEX* gene have more severe biochemical or skeletal phenotypes (Holm et al., 2001; Morey et al., 2011; Song et al., 2007). Other studies were unable to establish a genotype‐phenotype correlation when comparing patients with truncating variants to those with missense variants (Cho et al., 2005; Rafaelsen et al., 2016; Reid et al., 1989; Zhang et al., 2019). There are also multiple studies describing broad and clinically significant variations in XLH phenotype among patients with the same genotype, including among members of the same family (Holm et al., 2001; Rafaelsen et al., 2016; Rodríguez‐Rubio et al., 2021).

### 1.4 A new locus‐specific database for PHEX variants

Early and accurate diagnosis is beneficial for XLH patients as treatment leads to dramatic reduction in morbidities and improvement in patient quality of life. However, this is complicated by the rarity of the disease, phenotypic variability, similarities to other forms of congenital and sporadic hypophosphatemias, and the absence of a comprehensive source of *PHEX* disease‐associated variants needed for accurate and timely interpretation of genetic testing results.

A new locus‐specific database for *PHEX* gene variants, *PHEX* LSDB, was established to collect and disseminate information to the scientific community and affected families about disease‐causing *PHEX* variants (https://www.rarediseasegenes.com/). The database compiles variants from four sources: an older, archived *PHEX* locus‐specific variants database (Sabbagh et al., 2000), variants identified in a recent hypophosphatemia genetic testing program (Rush et al., 2021), unpublished variants identified in previous XLH clinical studies, and previously published variants identified from a comprehensive literature review. The *PHEX* LSDB will be updated regularly with new information on *PHEX* variants as reported in the literature or submitted directly to the database website. The purpose of this report is to describe the new *PHEX* LSDB and to present findings from an analysis of the initial set of variants in the database.

## 2 METHODS

### 2.1 Assembling the database

The database was developed by integrating *PHEX* variants from four different sources: (1) a now inactive McGill University *PHEX* locus‐specific database (Sabbagh et al., 2000), last updated April, 2017, (2) genetic testing results from burosumab clinical trials, (3) a comprehensive literature review, and (4) a sponsored hypophosphatemia genetic testing program initiated by Ultragenyx Pharmaceutical Inc. and Invitae Corporation, which provided no‐charge next‐generation sequencing with a multi‐gene panel to confirm a clinical XLH diagnosis or to aid diagnosis of suspected XLH or other genetic hypophosphatemia (Rush et al., 2021). The hypophosphatemia genetic testing program was designed to detect single nucleotide variants (SNV), small and large insertions/deletions (indels), sub‐genic structural variants, and exon‐level copy number variants (CNV).

A comprehensive literature review was performed using Mastermind, a database of variants with evidence cited in the medical literature (Genomenon Inc.) (Chunn et al., 2020) and considered all publications indexed in PubMed as of April 7, 2020. We followed stated guidelines for performing meta‐analyses from biomedical literature. Before completing variant curation, the data were cross‐checked against ClinVar and the McGill University *PHEX* database to ensure maximal sensitivity of the resulting data set. A complete accounting of the results of the literature curation process and data used for the American College of Medical Genetics (ACMG) interpretation process are provided in the supplemental methods.

Data from all four sources were assembled and manually reviewed to eliminate duplicates and ensure consistency in nomenclature and interpretation. Variants were annotated with interpretations and detailed clinical and biochemical phenotypes extracted from literature reports and clinical testing submission forms.

### 2.2 Patients and privacy

All individuals who provided samples for genetic testing, through the gene panel program or busosumab clinical trials, consented to have their deidentified genetic information published. To avoid accidental reidentification in this rare disease population, phenotypic data are reported in aggregate for each variant, and lab values are not reported for variants that occur fewer than three times in the database.

### 2.3 Variant nomenclature and classification

The nomenclature used in *PHEX* LSDB follows the Human Genome Variant Society (HGVS) guidelines (den Dunnen et al. 2016). Sequence variants were described using NCBI reference sequence NM_000444.6 for the *PHEX* gene and NP_000435.3 for the corresponding PHEX protein. Variant interpretations based on the consensus guidelines from the ACMG and the Association for Molecular Pathology are included, as reported by a CLIA certified clinical testing laboratory (“ACMG variant call”) or as predicted using variant interpretation software (“ACMG variant predictions”) (Nykamp et al., 2017; Richards et al., 2015). ACMG variant predictions were based on curations from the literature as well as data taken from multiple external databases for computational predictions (PolyPhen, MutationTaster, LoFtool, CAAD, and SIFT) and population frequencies (gnomAD).

### 2.4 Analysis

We used serum phosphorus values and reported clinical phenotypes to examine correlations between *PHEX* genotype with XLH phenotype. To control for age‐related variance, serum phosphorus values were analyzed as a percent of the lower limit of the provided normal ranges; values reported without an associated normal range were excluded from the analysis.

A 3‐D visualization of human PHEX protein was modeled based on homologous endopeptidases and using the PyMOL MolecularGraphics System, Version 2.4 (Schrödinger, LLC) with atomic coordinates from the Phyre2 web portal for protein modeling, prediction, and analysis (Kelley et al., 2015).

## 3 RESULTS

### 3.1 Spectrum of PHEX variants

As of April 30, 2021, the *PHEX* LSDB reports 2578 total XLH‐associated *PHEX* variants representing 870 unique variants (Table S1). The literature review data set contributed 223 unique variants that were not found in any other source, the genetic sequencing program contributed 204, burosumab clinical trial data contributed 65, the McGill data set contributed 22, and the remaining 356 unique variants were reported by more than one of the source datasets. Variants occur across the entire length of the gene, including deep within introns and in the 3′‐ and 5′‐UTR, with no apparent concentration of variants at one or more locations within the gene (Figure 2).

Figure 2Positions of 870 unique PHEX variants. (a) Variants that span multiple exons (copy number variants and complex rearrangements) are represented by lines above the exon map and intron‐specific variants are clustered by loci below. Del, deletion (grey lines); dup, duplication (blue lines). (b) Exon‐specific variants clustered by loci. Variants are indicated by affected nucleotides, affected amino acids, or affected exons, depending upon the limits of the detection techniques employed. ins, insertion; delins, combination deletion/insertion; >, substitution;  ?, nucleotide boundary as approximated by detection method. PHEX, Phosphate regulating gene with Homology to Endopeptidases that maps to the X chromosome

**Figure:**
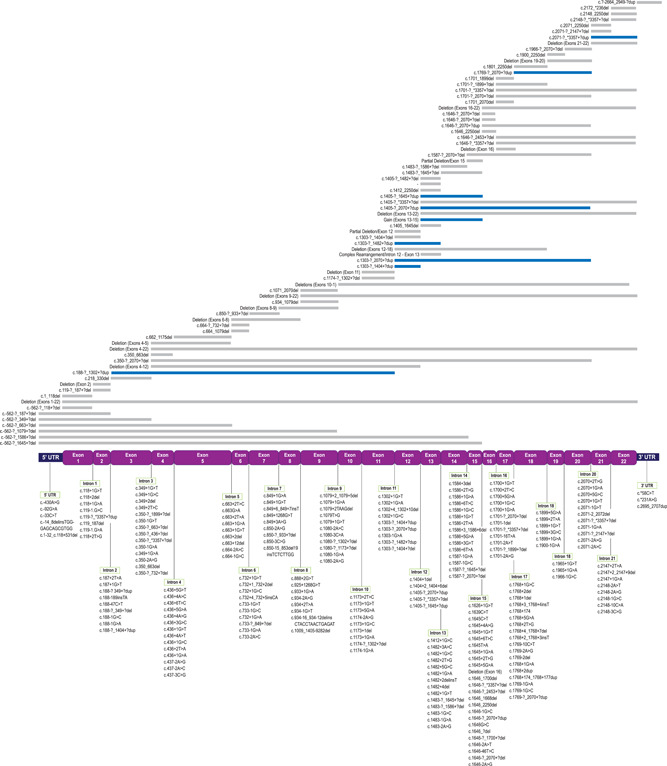

**Figure:**
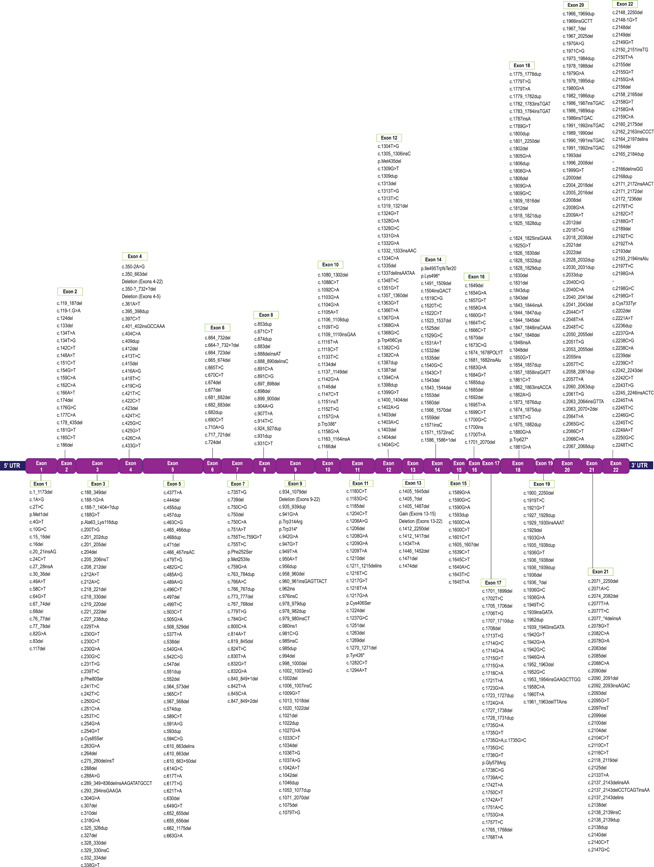

ClinVar reports 814 XLH‐related variants in *PHEX* (accessed May 24, 2021), a recently updated count which includes 287 variants identified in the hypophosphatemia genetic testing program and reported to ClinVar (Rush et al., 2021). The locus‐specific database contributes another 50 novel XLH‐related *PHEX* variants which are presented here for the first time (Table S2).

The most prevalent variant in the database is a c.*231A>G substitution in the 3′‐UTR, which was frequently reported co‐occurring with an exon 13–15 duplication, suggesting that these two variants co‐segregate and constitute a single allele. In an analysis reported from the hypophosphatemia gene panel program, these variants were found together in both males and females in 65 of 66 probands and were shown to be in cis in all 51 individuals for whom phasing information was available (Rush et al., 2021).

**Figure 3:**
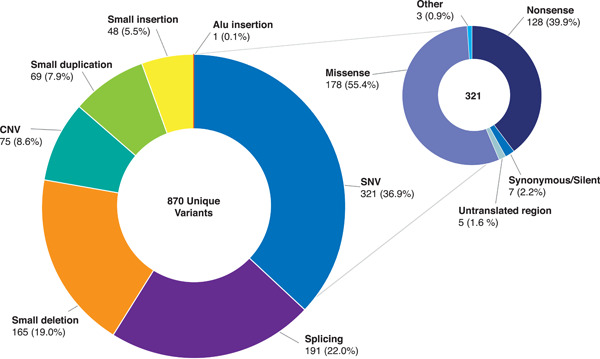
Distribution of 870 unique PHEX variants by variant type. CNV, copy number variant (size greater than 100 nucleotides [nt]); small deletion (size smaller than 100 nt); small duplication (size smaller than 100 nt); small insertion (size smaller than 100 nt); SNV, single nucleotide variant. One allele that contains two variants (Ex13–15 dup and c.*231A>G) was counted in both the CNV and SNV categories. “Other” included 1 start codon change and 1 deep intronic variant. PHEX, Phosphate regulating gene with Homology to Endopeptidases that maps to the X chromosome

Single nucleotide variants (SNV) represent the most common type of unique variant in the database (*n* = 321, 36.9%) with the majority causing missense or nonsense protein variations (*n* = 178 and 128, respectively). Splicing variants (*n* = 191, 22.0%) are the second most common type followed by small deletions (*n* = 165, 19.0%), copy number variants (CNV) (*n* = 75, 8.6%), small duplications (*n* = 69, 7.9%), and small insertions (*n* = 48, 5.5%) (Figure 3). The relative frequency (most common to least common) of each variant type is the same when considering all 2578 (nonunique) variants in the database: SNV (*n* = 1283, 49.8%); splicing (*n* = 530, 20.6%); small deletions (*n* = 305, 11.8%); CNV (*n* = 293, 11.4%); small duplications (*n* = 98, 3.8%); and small insertions (*n* = 68, 2.6%).

Individuals represented in the database have a broad geographical distribution encompassing North America, Europe, Middle East, and Asia. Ten of the 12 most common variants were found across multiple geographies while the most common bi‐variant allele, c.*231A>G 3’‐UTR + Ex13–15 duplication, was found only in North America to date (Table 1).

**Table 1:** Geographic distribution of the 11 most common alleles in *PHEX* LSDB

Variant	Number of occurrences (range)	North America	Europe	Asia	Middle East	Turkish	Unspecified
c.1405‐?_1645+?dup/c.*231A>G	>30	Yes	No	No	No	No	No
c.1601C>T	>30	Yes	Yes	Yes	No	Yes	Yes
c.1645+1G>A	>30	Yes	Yes	Yes	No	Yes	Yes
c.1645C>T	>30	Yes	Yes	Yes	No	Yes	Yes
c.1699C>T	>30	Yes	Yes	Yes	No	No	Yes
c.1735G>A	>30	Yes	Yes	Yes	No	Yes	Yes
c.2104C>T	>30	Yes	Yes	Yes	No	Yes	Yes
c.2239C>T	>30	Yes	Yes	Yes	Yes	Yes	Yes
c.871C>T	>30	Yes	Yes	Yes	No	No	Yes
c.1209G>A	11–20	Yes	Yes	Yes	No	No	Yes
c.58C>T	11–20	Yes	Yes	Yes	No	No	Yes

*Note*: Geography is based on location of study site or patient ethnicity, as provided in the source data.

### 3.2 Clinical significance and biological relevance of PHEX variants

ACMG variant calls assigned by a CLIA certified laboratory were reported for 469 of the 870 variants in the *PHEX* LSDB, with the majority categorized as pathogenic or likely pathogenic (*n* = 338 + 60, 84.8%) and the remaining categorized as variants of uncertain significance (VUS; *n* = 71, 15.2%). An additional 401 variants from research studies in the literature were assigned an ACMG category using variant interpretation software. The most common predicted ACMG category was pathogenic (*n* = 242, 60.3%) followed by VUS (*n* = 96, (23.9%), likely pathogenic (*n* = 57, 14.2%), and benign/likely benign (*n* = 6, 1.5%).

Six hundred seventy‐four disease‐associated *PHEX* variants cause premature stop codons, exon‐level copy number changes, or splicing defects, providing a clear prediction for biological loss of function. Conversely, 178 (20.5%) cause missense substitutions, which lead to minimal changes to the protein‐coding sequence making it difficult to predict the biologic effect. However, some of the missense variants were associated with published functional studies that demonstrated protein loss of function. Twelve different missense variants, p.Cys77Tyr, p.Cys85Arg, p.Cys85Ser, p.Ile281Lys, p.Ile333del, p.Ala514Pro, p.Gly572Ser, p.Gly579Arg, p.Gly579Val, p.Ser711Arg, p.Ala720Thr, and p. Phe727Leu, all produced recombinant proteins that were not terminally glycosylated and remained trapped inside transformed cells (Li et al., 2020; Sabbagh et al., 2001, 2003; Zheng et al., 2020). In the same studies, missense variants p.Asp237Gly, p.Tyr317Phe, p.Gly553Glu, and p.Phe731Tyr were efficiently transported to the cell membrane but showed substantially reduced enzymatic activity or altered protein conformation (Sabbagh et al., 2003; Zheng et al., 2020).

Even in the absence of data from animal models or in vitro assays, the position of missense variants can sometimes provide clues about their potential impact on protein function. The database includes 28 unique missense variants at nine different residues forming disulfide bonds (Cys59, Cys77, Cys85, Cys142, Cys406, Cys617, Cys693, Cys733, and Cys746), which could lead to protein conformation/trafficking defects, and two missense variants that occur at a glycosylation site (Asn71), which could lead to defects in protein trafficking. There are also four missense variants at zinc coordination sites (His580 and His584) and one missense variant at each of the active site residues (Glu581, Asp646), which are likely to disrupt enzymatic activity.

Consistent with their potential biological relevance, most variants mentioned in this section have an ACMG variant call or predicted call of pathogenic or likely pathogenic. The remaining eleven variants (p.Cys59Ser, p.Cys142Arg, p.Cys142Ser, p.Cys617Phe, p.Cys733Ser, p.Cys733Phe, p.Cys746Trp, p.Asn71Thr, p.His580Pro, p.His584Pro, and p.Asp646Asn) have a variant call or predicted call of VUS.

### 3.3 Phenotypic range of disease presentation and genotype/phenotype analysis

When reported, the most common abnormal phenotypes associated with the variants were: lower limb deformities (reported for 405 variants), other musculoskeletal abnormalities (reported for 166 variants), gait abnormalities (reported for 148 variants), tooth abscesses and/or excessive dental carries (reported for 142 variants), short stature (reported for 104 variants), and fractures/pseudofractures (reported for 75 variants).

Evaluable serum phosphorus values were reported for 224 individuals representing 128 variants. We found no significant variance in the average % lower limit of normal (LLN) phosphorus level when comparing values for: the full analysis group (74% [*n* = 224, SD = ±16]); the full group excluding c.*231A>G/Ex13–15 dup (73% [*n* = 187, SD = ±15]); the 11 most common alleles (74% [*n* = 78, SD = ±16]); the c.*231A>G/Ex13–15 dup allele (72% [*n* = 41, SD = ±13]); and the remaining 10 most common alleles excluding c.*231A>G/Ex13–15 dup, (72.0% [*n* = 41, SD = ±13]) (Table 2). Females had higher mean serum phosphorus levels compared with males in each of these groups, but this elevation was not found to be statistically significant.

**Table 2:** Relative serum phosphorus levels from 128 variants with evaluable data

Group	Average %LLN Phosphorus (SD) [range]		
	Full group	Females	Males
All variants with evaluable data^a^	74% (±16) [3%–100%] *n* = 224	77% (±15) [23%–100%] *n* = 135	69% (±16) [3%–100%] *n* = 87
All variants without c.*231A>G	73% (±15) [23%–100%] *n* = 187	76% (±15) [23%–100%] *n* = 116	69% (±14) [34%–100%] *n* = 69
Top 12 variants^b^	74% (±16) [3%–100%] *n* = 78	82% (SD = ±12) [33%–1 00%] *n* = 38	67% (±16) [3%–100%] *n* = 40
Top 12 Variants^b^ without c.*231A>G	72% (±13) [40%–100%] *n* = 41	81% (SD = ±9) [60%–96%] *n* = 19	64% (±11) [40%–85%] *n* = 22
c.1405‐?_1645+?dup/c.*231A>G	77% (±19) [3%–100%] *n* = 37	83% (±15) [33%–100%] *n* = 19	71% (±21) [3%–100%] *n* = 18

Abbreviations: LLN, lower limit of normal range; *n*, number of evaluable phosphate levels; SD, standard deviation.

^a^ Phosphorus values were reported with an associated normal range for 128 variants; values reported without respect to a normal range were excluded from the analysis.

^b^ Twelve most common variants in PHEX LSDB represent 11 alleles and occurred >15 times.

We also examined serum phosphorus levels for the 11 most common *PHEX* alleles, which occurred at least 15 times in the database. Only nine of these alleles had one or more phosphorus levels reported with respect to a normal range, and we found no statistically significant differences in the relative phosphorus values between these groups (Table 3). The highest average phosphorus level in this analysis was 92% LLN for c.1645 + 1 G>A (*n* = 2, females) indicating relatively mild hypophosphatemia. In contrast, this splicing variant has a pathogenic ACMG call and the reported clinical phenotypes, which include teeth falling out, lower limb deformities/bowing of legs, severe skeletal phenotype, and fractures/pseudofractures, could be indicative of moderate or severe disease.

**Table 3:** Relative serum phosphorus levels from patients harboring most common *PHEX* alleles

**Variant**	**Predicted effect**	**ACMG categories**	**Average %LLN Phosphorus** a **(SD) [range]**		
			Full group	Females	Males
c.1405‐?_1645+?dup/c.*231A>G	frameshift/Ex13‐15dup	Likely pathogenic (both)	77% (±19) [3%–100%] *n* = 37	83% (±15) [33%–100%] *n* = 19	71% (±21) [3%–100%] *n* = 18
c.1601C>T	p.Arg567*	Pathogenic	74% (±9) [61%–88%] *n* = 9	83% (±4) [76%–88%] *n* = 4	66% (±5) [61%–76%] *n* = 5
c.1645+1G>A	Splice donor	Pathogenic	92% (±4) [88%–96%] *n* = 2	92% (SD = ±4) (*n* = 2) [88%–96%]	n/a
c.1645C>T	p.Arg549*	Pathogenic	62% (±13) [40%–76%] *n* = 5	66% (±7) [60%–76%] *n* = 3	57% (±17) [40%–74% *n* = 2
c.1699C>T	p.Arg567*	Pathogenic	74% (±10) [60%–83%] *n* = 3	81% (±2) [79%–83%] *n* = 2	60% *n* = 1
c.1735G>A	p.Gly579Arg	Pathogenic	74% (±11) [57%–89%] *n* = 10	82% (±6) [72%–89%] *n* = 6	63% (±7) [57%–74%] *n* = 4
c.2104C>T	p.Arg702*	Pathogenic	53% (±4) [49%–57%] *n* = 2	n/a	53% (±4) [49%–57%] *n* = 2
c.2239C>T	p.Arg747*	Pathogenic	75% (±7) [66%–85%] *n* = 4	n/a	75% (±7) [66%–85%] *n* = 4
c.871C>T	p.Arg291*	Pathogenic	70% (±16) [50%–93%] *n* = 6	88% (±5) [83%–93%] *n* = 2	61% (±11) [50%–76%] *n* = 4

*Note*: These 9 alleles occurred >15 times in the database and represent 10 variants; 2 additional variants that occurred >15 times (c.1209G>A and c.58C>T) had no phosphate values reported with respect to a reference range.

Abbreviations: LLN, lower limit of normal range; *n*, number of evaluable phosphate levels; n/a, no evaluable phosphorus values available.

^a^ Phosphorus values reported without respect to a reference range were excluded.

### 3.4 The PHEX LSDB website

This *PHEX* LSDB is openly available to the greater scientific and XLH patient communities through a public website, https://www.rarediseasegenes.com/. Key components of the website are the main variant table and interactive data pages displaying summary variant types, genotype‐phenotype data, and summary geographical data reported for the variants.

**Figure 4:**
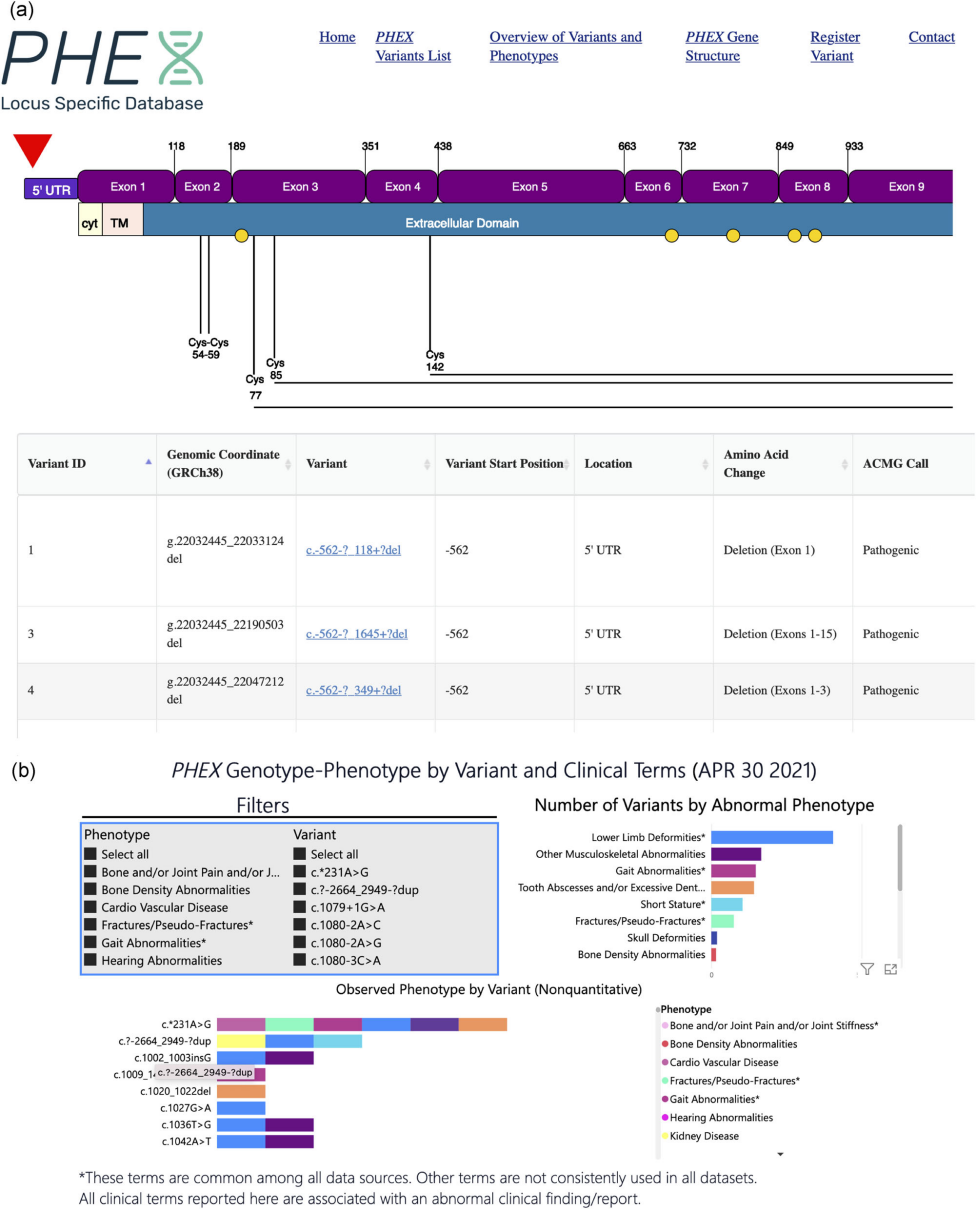
Screenshots from the PHEX LSDB website. (a) The main variant table provides specific information on all variants identified in the database including HSGV variant nomenclature, predicted effects, ACMG variant prediction, and all publications reporting the variant. The red arrow, above the exon and protein maps at the top of the page, indicates the position of a selected variant. (b) An interactive display of genotype‐phenotype analysis by variant and abnormal phenotype

The main variant table provides specific information on all variants identified in the database including Human Genome Variation Society (HSGV) variant nomenclature, predicted effects, ACMG variant prediction, and all publications reporting the variant (Figure 4a). The table can be searched by any of the fields listed in Table 4 and the position of a selected variant relative to structural features of the gene and protein is displayed above the table. The interactive data pages allow users to explore genotype‐phenotype correlations using biochemical data (serum phosphorus, TmP/GFR, and bone‐specific alkaline phosphatase) or reported clinical phenotypes (Figure 4b).

**Table 4:** *PHEX* LSDB variant table fields

Field	Description
Variant ID	The ID number assigned by the database to a specific variant
Variant	cDNA changes in ACMG standard format, based on transcript NM_000444.6
Variant Start Position	Numbered position in the coding sequence of the gene where the variant starts, based on transcript NM_000444.6
Location	Region of the gene or surrounding sequence where the variant starts
Amino Acid Change	Amino acid change associated with the cDNA change in standard ACMG format
ACMG Call	Interpretation of the variant in ACMG format (e.g. pathogenic, likely pathogenic, variant of uncertain significance/VUS) as reported by a CLIA‐certified clinical testing laboratory
Last Revised Date	Date the variant was submitted or date of last update to the variant classification provided to website.
Predicted ACMG Call	Interpretation of the variant based on variant prediction software or as reported in published literature
Variant Type	Gene variants are described according to the Variant Type, which include: CNV, copy number variant which here describes a change 100 DNA nucleotides or larger; SNV, single DNA nucleotide variant; small insertion; splicing, affects how RNA is made; small deletion; and small duplication, and Alu, insertion of an Alu repeat element.
Effect Type	Effect type can be: Deletion, Untranslated region, Frameshift, Missense, Nonsense, Synonymous/Silent, Splice donor, Splice acceptor, Duplication, Intronic, Insertion, and Deletion/Insertion.
Times Observed	The number of times a variant has been observed in patients. To protect privacy, individual patient counts are not provided.
PMID	The PubMed ID for a published article associated with the variant.
Article Count	The number of PubMed articles associated with the variant.
Clinical and Biochemical Phenotype	Qualitative descriptions of clinical and biochemical signs and symptoms (phenotypes) reported for all patients with the variant. Numerical laboratory values are reported separately in the Overview of variants and associated phenotypes pages. To protect privacy, phenotypes are not reported for variants that occur 3 or fewer times in the database.

The *PHEX* LSDB encourages submission of novel *PHEX* variants as well as new patient data for variants that are already reported. The online submission form, available on the website, collects the submitters contact information and detailed information on the variant, including cDNA change, amino acid change, ACMG call and date of interpretation, allele origin, clinical and biochemical phenotypes, geography, and any associated PubMed identifier (PMID). Submitters are required to obtain appropriate consent for data contribution and deidentify all patient data before submission.

## 4 DISCUSSION

By combining information from the older McGill *PHEX* variant database, variants from the sponsored hypophosphatemia genetic testing program, previously unpublished Ultragenyx clinical study variants, and a comprehensive literature review of published variants, we describe the largest collection of unique *PHEX* variants to date, for a total of 870. *PHEX* does not appear to have a predominant variant and many variants were only described one or a few times in the database. Variants occur across the entire *PHEX* gene and examining the position of variants did not identify any apparent concentration of variants or obvious protein binding sites. Although the overall pattern of variants is consistent with previous studies, there has been a large increase in the number of CNV reported, which is likely a reflection of improved genetic testing methods for CNV. In the hypophosphatemia genetic testing program, 81% of the CNV events detected were sub‐genic and would be missed by traditional microarray analysis (Rush et al., 2021). However, despite the increased detection of CNV from more recent reports, most of the routine clinical testing methods employed so far, including those used in the hypophosphatemia genetic testing program, cannot detect deep intronic variants or large structural variants with breakpoints outside of the *PHEX* coding region.

There are documented cases/family cohorts of clinically confirmed XLH for which no variant has been detected with the genetic tests employed to date. In the hypophosphatemia genetic testing program, approximately 10% of clinically confirmed XLH patients had no variant identified in *PHEX* or in another gene, suggesting that up to 10% of clinical XLH patients may have an undetected *PHEX* variant (Rush et al., 2021). This underscores the utility of genetic testing methods that can detect sub‐genic CNV, deep intronic variants, and large structural variations to establish a molecular diagnosis for XLH. Such methods include RNA sequencing and whole‐genome sequencing with computational analysis to detect intronic and structural variants.

The most common variant in the database is the bi‐variant allele, c.*231A>G, Ex13–15 dup, which has been described previously and reported to be specific to patients from the Midwest region of the United States (Mumm et al., 2019). The expanded data from the hypophosphatemia genetic testing program revealed that these two variants occur together across the United States in individuals of White/Caucasian, Hispanic, and Black/African American ancestries (Rush et al., 2021). This is also consistent with earlier findings of XLH families from outside of the US Midwest geography carrying the *231A>G variant (Ichikawa et al., 2008).

Earlier, small cohort studies identified c.*231A>G as a disease‐causing variant in multiple probands leading to XLH (Ichikawa et al., 2008; Smith et al., 2020), however, these studies did not detect CNV, so it is not known whether these patients also carried the Ex‐13–15 duplication. The recent readout from the hypophosphatemia genetic testing program found that all XLH patients who had the 3′‐UTR c.*231A>G variant also carried the Ex13–15 duplication, but one affected proband carried the duplication without the c.*231A>G variant, suggesting that the duplication can contribute to disease on its own (Rush et al., 2021). In the *PHEX* LSDB, which includes all the variants described by Rush et al., age‐normalized relative phosphorus levels associated with the c.*231A>G, Ex13–15 dup variant were not significantly different than those from all other variants, adding to the mounting evidence that this bi‐variant allele leads to clinically significant disease. Although the evidence cited above suggests that the Ex 13–15 duplication is disease‐causing on its own, further data are needed to determine whether c.*231A>G leads to a disease phenotype when it is the only *PHEX* variant present.

There was a lack of identifiable genotype‐phenotype correlation regarding serum phosphorus. Somewhat surprisingly there is not a significant variation in phenotype observed for active site variants and variants that have an early stop codon have a similar phenotype to variants that occur later in the gene, suggesting that the mutant PHEX proteins resulting from these different categories of variants have a similar pathophysiological effect. As described here, the pathogenicity of certain *PHEX* missense variants has been demonstrated with functional assays (Li et al., 2020; Sabbagh et al. 2001, 2003; Zheng et al., 2020).

### 4.1 XLH management and treatment

Historically, the standard of care for XLH patients has been a combination of calcitriol and phosphate supplementation, which may improve bone mineralization by providing transient incremental elevations in phosphate administered multiple times daily (Carpenter et al., 2011; Dahir et al., 2020). Recently, the novel therapeutic burosumab, a fully human monoclonal antibody against FGF23, was approved for the management of pediatric and adult patients with XLH (Imel et al., 2019; Insogna et al., 2018). In randomized clinical studies, burosumab, which binds FGF23, resulted in increased reabsorption of phosphate in the kidney and increased serum phosphorus levels. Treatment, especially when initiated early in the course of disease, can provide substantial improvements in outcomes. Therefore, accurate early diagnosis of XLH helps to provide prognostic information, inform patients about the need for lifelong disease management, and guide medical therapy. This comprehensive searchable database of *PHEX* variants aggregates and organizes the evidence around variants for medical, scientific, and lay audiences, enabling earlier diagnosis and better patient outcomes.

### 4.2 Limitations

We were not able to determine reliably whether clinical and biochemistry data provided in case reports or genetic testing forms is treatment naïve or not. There was limited evaluable biochemical data because the age of patients was not always known, and lab values were frequently provided without reported age‐specific reference ranges. Clinical data were also extremely limited in this data set, and we were not able to quantitatively evaluate genotype/phenotype correlations for skeletal or other non‐biochemical outcomes. In addition, it was not always possible to know when patients were related or whether data for the same individual was reported by more than one molecular diagnostics lab. For this reason, allele frequency determinations from this data set cannot reliably be made.

### 4.3 PHEX LSDB curation and updates

The *PHEX* LSDB is overseen and curated by a qualified scientific committee. New *PHEX* variants originating from publications, online submissions, or genetic testing labs will be added regularly. Following review by the scientific committee, all data displays and analysis pages will be updated accordingly, and new variants will be shared twice‐yearly with ClinVar and the Leiden Open Variation Database.

## CONFLICT OF INTERESTS

This study was sponsored and funded by Ultragenyx Pharmaceutical Inc. (Ultragenyx) in partnership with Kyowa Kirin International. Y. Sabbagh is an employee of Inozyme Pharma. S. Daugherty, N. Miller, S. Krolczyk and P. Ramesan are employees and shareholders of Ultragenyx. S. Sarafrazi, P. Boada and S. Eisenbeis are former employees of Ultragenyx and S. Eisenbeis is also a shareholder. K. Dill is a 3rd party contractor for Ultragenyx. B. Johnson and R. Truty are employees and shareholders of Invitae Corporation. L. Chunn and M. J. Kiel are employees of Genomenon Inc. T. O. Carpenter and E. A. Imel have received research funding from Ultragenyx. M. J. Econs has received consultant fees from Ultragenyx and holds a patent that is licensed to Ultragenyx.

## WEB RESOURCES

PolyPhen: http://genetics.bwh.harvard.edu/pph2/

MutationTaster: https://www.mutationtaster.org/

LoFtool and CAAD are plugins developed by Ensembl and available in a public Github repository: https://github.com/Ensembl/VEP_plugins

SIFT: https://sift.bii.a-star.edu.sg/

gnomAD: https://gnomad.broadinstitute.org/

Phyre2 web portal: http://www.sbg.bio.ic.ac.uk/phyre2/html/page.cgi?id=index

Liden Open Variation Database: https://www.lovd.nl/

ClinVar: https://www.ncbi.nlm.nih.gov/clinvar

## Supporting information

Supporting information.

Supporting information.

## Data Availability

The *PHEX* LSDB reports data in aggregate for each variant; it is available at https://www.rarediseasegenes.com/ and has also been submitted to the Liden Open Variation Database (LOVD) (https://www.lovd.nl/). All novel PHEX variants reported here for the first time have also been submitted to ClinVar (https://www.ncbi.nlm.nih.gov/clinvar/).
